# ARG1 as a promising biomarker for sepsis diagnosis and prognosis: evidence from WGCNA and PPI network

**DOI:** 10.1186/s41065-022-00240-1

**Published:** 2022-06-23

**Authors:** Jing-Xiang Zhang, Wei-Heng Xu, Xin-Hao Xing, Lin-Lin Chen, Qing-Jie Zhao, Yan Wang

**Affiliations:** School of Pharmacy, Second Military Medical University, Shanghai, 200433 China

**Keywords:** Sepsis, Bioinformatical analysis, Differentially expressed genes, WGCNA, ARG1

## Abstract

**Background:**

Sepsis is a life-threatening multi-organ dysfunction caused by the dysregulated host response to infection. Sepsis remains a major global concern with high mortality and morbidity, while management of sepsis patients relies heavily on early recognition and rapid stratification. This study aims to identify the crucial genes and biomarkers for sepsis which could guide clinicians to make rapid diagnosis and prognostication.

**Methods:**

Preliminary analysis of multiple global datasets, including 170 samples from patients with sepsis and 110 healthy control samples, revealed common differentially expressed genes (DEGs) in peripheral blood of patients with sepsis. After Gene Oncology (GO) and pathway analysis, the Weighted Gene Correlation Network Analysis (WGCNA) was used to screen for genes most related with clinical diagnosis. Also, the Protein-Protein Interaction Network (PPI Network) was constructed based on the DEGs and the hub genes were found. The results of WGCNA and PPI network were compared and one shared gene was discovered. Then more datasets of 728 experimental samples and 355 control samples were used to prove the diagnostic and prognostic value of this gene. Last, we used real-time PCR to confirm the bioinformatic results.

**Results:**

Four hundred forty-four common differentially expressed genes in the blood of sepsis patients from different ethnicities were identified. Fifteen genes most related with clinical diagnosis were found by WGCNA, and 24 hub genes with most node degrees were identified by PPI network. *ARG1* turned out to be the unique overlapped gene. Further analysis using more datasets showed that *ARG1* was not only sharply up-regulated in sepsis than in healthy controls, but also significantly high-expressed in septic shock than in non-septic shock, significantly high-expressed in severe or lethal sepsis than in uncomplicated sepsis, and significantly high-expressed in non-responders than in responders upon early treatment. These all demonstrate the performance of *ARG1* as a key biomarker. Last, the up-regulation of *ARG1* in the blood was confirmed experimentally.

**Conclusions:**

We identified crucial genes that may play significant roles in sepsis by WGCNA and PPI network. *ARG1* was the only overlapped gene in both results and could be used to make an accurate diagnosis, discriminate the severity and predict the treatment response of sepsis.

**Supplementary Information:**

The online version contains supplementary material available at 10.1186/s41065-022-00240-1.

## Introduction

Sepsis is defined as a severe systemic organ dysfunction due to a dysregulated host response to infection [1]. More than 30 million people are effected annually worldwide [2]. According to a meta-analysis which reviewed 170 studies around the world, the 90-day mortality of sepsis patients was 32.24% (95% CI 27.0–37.5%) [3]. However, up to now, we still do not fully understand the pathogenesis of sepsis, and are lack of specific drugs. Treatment for sepsis depends mainly on supportive measures, and patients at the early stage showed the best response [4]. Therefore, it is important to recognize sepsis early, so that supportive measures may be implemented as soon as possible.

Different biomarkers have been used for diagnosis of sepsis and monitoring of treatment, such as proclcitonin (PCT), C-reactive protein (CRP), cytokines, and human leukocyte antigen DR (HLA-DR) [5–12]. Although these biomarkers are widely employed in clinical practice for monitoring the infectious process or inflammatory disorders, none of them has sufficient specificity to distinguish sepsis from other inflammatory disorders. So there still demands a novel biomarker to provide valuable information for specific diagnosis of sepsis.

**Fig. 1:**
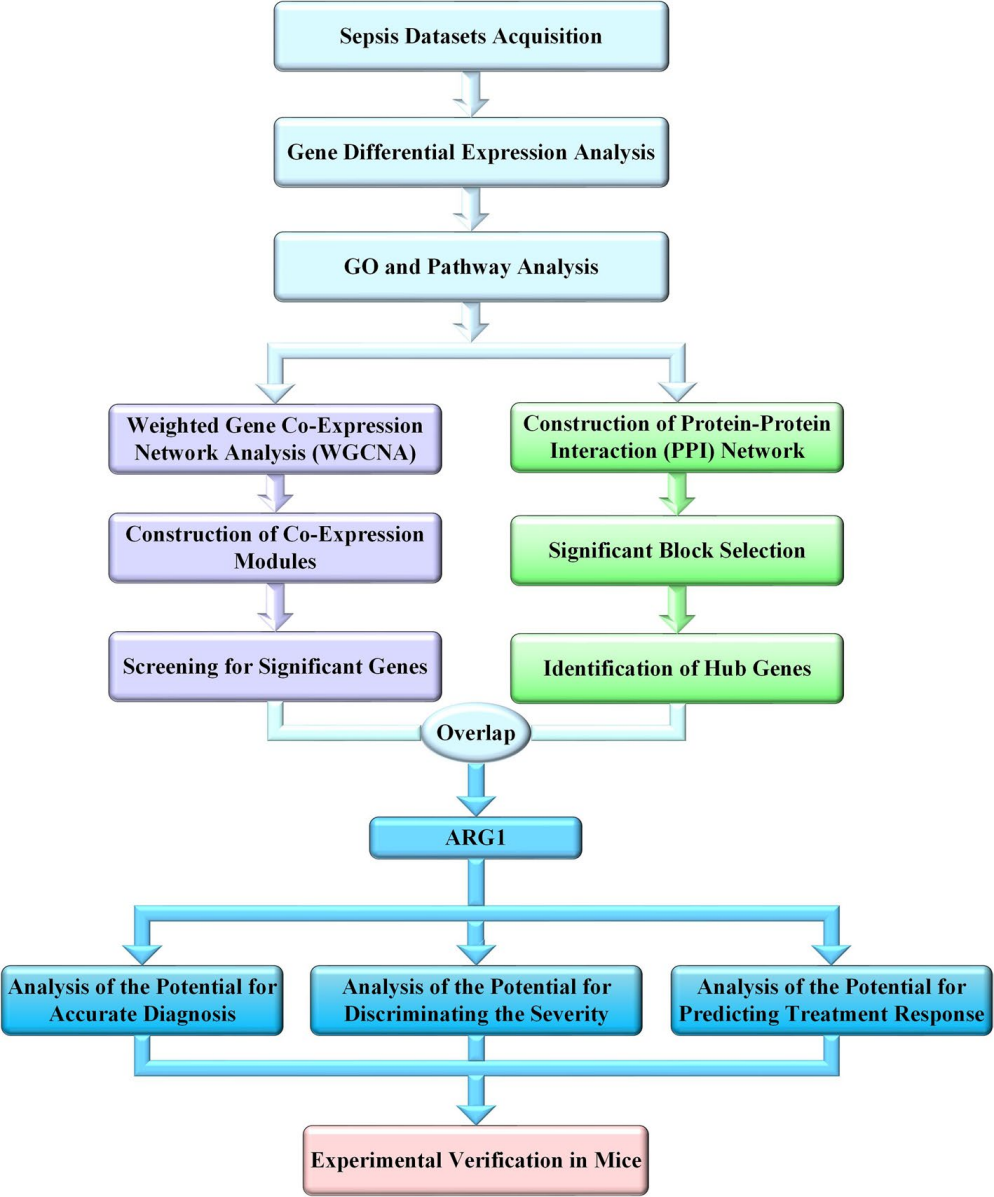
The work flow chart of this study

In recent decades, genome-wide analyses, such as high-throughput sequencing technology and gene chips, have been routinely used to study gene expression patterns [13]. The analysis of these data has fostered the development of bioinformatics and derived methods such as Weighted Gene Co-Expression Network Analysis (WGCNA) and Receiver Operating Characteristic (ROC) analysis, which can provide valuable information for biomarker discovery of diseases [14–16]. In this study, we used the gene expression datasets from different populations in different countries to screen out the common differentially expressed genes (DEGs) in peripheral blood cells of patients with or without sepsis. After functional enrichment analyses, WGCNA was conducted to identify genes highly associated with clinical traits. Parallelly, the PPI network was constructed to identifiy genes which may be involved in the progression of sepsis. Then, the two groups of screened-out genes were compared and the promising biomarker gene of sepsis was revealed. Moreover, a series of additional datasets were then used to prove the diagnosis and prognosis role of this gene. Last, the bioinformatic results were confirmed experimentally by real-time PCR. The overall design of this study was shown as a flow chart in Fig. 1.

## Materials and methods

### Data collection

Datasets of gene expression profiles GSE8121, GSE13015, GSE26378, GSE26440, GSE28750, GSE57065, GSE60424, GSE63042, GSE65682, GSE69528, GSE95233, GSE110487, GSE131411, GSE131761, GSE134347, GSE145227 and GSE154918 were downloaded from the NCBI-GEO Datasets Database. Details of the datasets were shown in Supplementary Table 1.

### DEGs identification, Gene Ontology (GO) and pathway enrichment analysis

Data pre-processing and gene expression profile comparison between sepsis patients and controls were implemented by limma R package. Statistically significant DEGs were defined with adjusted *p* value < 0.05 and |logFC| > 1 as the cut-off criterion. Venny v2.1 was used to seek for the common DEGs from the four sample groups.

Database for Annotation, Visualization and Integrated Discovery (DAVID) was used for Gene Ontology (GO) analysis of the DEGs [17]. KEGG Orthology Based Annotation System (KOBAS) was used for pathway enrichment analysis [18]. *P* < 0.05 was considered statistically significant for GO and pathway enrichment analysis.

### Construction of weighted gene co-expression network and detection of modules

Weighted Gene Co-Expression Networks Analysis (WGCNA) is an R package which can construct a gene co-expression network from a large number of genes and identify co-expression modules [19]. The expression matrixes of DEGs identified above were meta-analyzed by WGCNA algorithm. First, the gene expression profiles of samples were clustered to remove the outliers. Then the soft threshold for network construction was selected, which maked the adjacency matrix to be the continuous value between 0 and 1, so that the constructed network conformed to the power-law distribution and was closer to the real biological network state [20]. Finally, the scale-free network was constructed using the blockwise modules function, followed by the module partition analysis to identify gene co-expression modules based on topological overlap, which could group genes with similar patterns of expression.

### Identifying clinically related modules and genes

All modules were summarized by module eigengenes (MEs), the foremost principle component of each module that was calculated as a synthetic gene representing the expression profile of all genes within a given module [21]. And the correlation between MEs and clinical traits (diagnosed sepsis or healthy) was computed. Then the gene-trait significance value (GS), which represented the relative level between genes and traits, were calculated by the value of Pearson’s correlation in order to identify genes most associated with sepsis [22].

### Construction of protein-protein interaction (PPI) network and identification of hub genes and blocks

Search Tool for the Retrieval of Interacting Genes/Proteins (STRING 11.0) is a database for protein-protein interactions [23]. In this study, we used STRING to map the identified common DEGs into the human PPI network, which was then visualized by Cytoscape. Number of interactions between each gene in the network were calculated using the Network Analyzer plug-in of Cytoscape [24], and those with more than 35 interactions were determined as hub genes. Furthermore, the Molecular Complex Detection (MCODE) plug-in was utilized to select the significant blocks from the PPI network, with cut-off criteria of degree ≥2, node score ≥ 0.2, K-core ≥2, and max depth = 100.

### Receiver Operating Characteristic (ROC) curve analysis

ROC curve analyses were performed with the use of R package pROC, which is open-sourced and often utilized to evaluate biomarker performances. The ROC curves were plotted based on specificity and sensitivity, and the AUC value was applied to predict diagnostic values of the selected gene.

### Animals

Female C57BL/6 mice (6–8 weeks old) were purchased from Shanghai SLAC Laboratory Animal Company (Shanghai, China). The mice were reared at 18 °C–22 °C and the humidity was about 55%. Mice were given light and dark environments according to their circadian rhythms. In addition, they were given free food and water.

### Cecal Ligation and Puncture (CLP) -induced sepsis

Poly-microbial sepsis was induced by Cecum Ligation and Puncture (CLP) surgery as previously described [25]. Briefly, preoperative fasting for 12 hours, mice were intraperitoneally injected for anaesthetization. Hair in the lower quadrant of the abdomen was removed with a depilation cream and the area was disinfected with 75% alcohol. Then a midline incision was made to obtain access to the peritoneal cavity, and the cecum was exposed, ligated, and punctured with a 22-gauge needle. Then, the ligated cecum was compressed slightly to squeeze out some cecal content. Next, the ligated cecum was slightly squeezed to squeeze out a small amount of cecal contents. The cecum was put back to its normal location, and incisions were closed. Sham-operated (control) animals underwent anaesthetization, incision, exposure and suture, but without cecal ligation and puncture. Postoperative incision was disinfected with iodophor. Mice were given normal saline at 37 °C (5 mL per 100 g body weight) subcutaneously for resuscitation. Besides, appropriate measures were taken to alleviate the postoperative pain of mice. The sample size of each group was 7.

### Quantitative real-time PCR

Peripheral blood of each mice was collected after 24 hours of CLP or Sham operation, and was treated with Red Blood Cell Lysis Buffer (Biosharp, Anhui, China). Total RNA was extracted from blood cells by RNA Fast 200 kit (Fastagen, Shanghai, China). Then reverse transcription was performed with the PrimeScript RT reagent Kit with gDNAEraser (Takara, Tokyo, Japan). Quantitative real-time PCR was conducted with the SYBR Green PCR kit (Yeasen, Shanghai, China) and StepOnePlus Real-Time PCR System (Thermo Scientific, Massachusetts, United States). Glyceraldehyde-3-phosphate dehydrogenase (GAPDH) was used as an internal control. The primer sequences of *ARG1* and *GAPDH* were as following: *ARG1*: forward TCACCTGAGCTTTGATGTCGA; reverse TGAAAGGAGCCCTGTCTTGTA. *GAPDH*: forward TCACCATCTTCCAGGAGCGAGAC; reverse AGACACCAGTA GACTCCACGACATAC. The results were analyzed by Mann-Whitney U test.

## Results

### DEGs identification, Gene Ontology (GO) and pathway enrichment analysis

The GEO database was utilized to obtain gene expression profile datasets in peripheral blood of septic patients. Four datasets (GSE28750, GSE57065, GSE65682 and GSE69528) representing different populations from Australia, France, Malta and United States were first obtained from the GEO database. The number of sepsis samples in GSE28750, GSE57065, GSE65682 and GSE69528 was 10, 26, 51 and 83 respectively, and the number of control samples was 20, 25, 42 and 28 respectively.

**Fig. 2:**
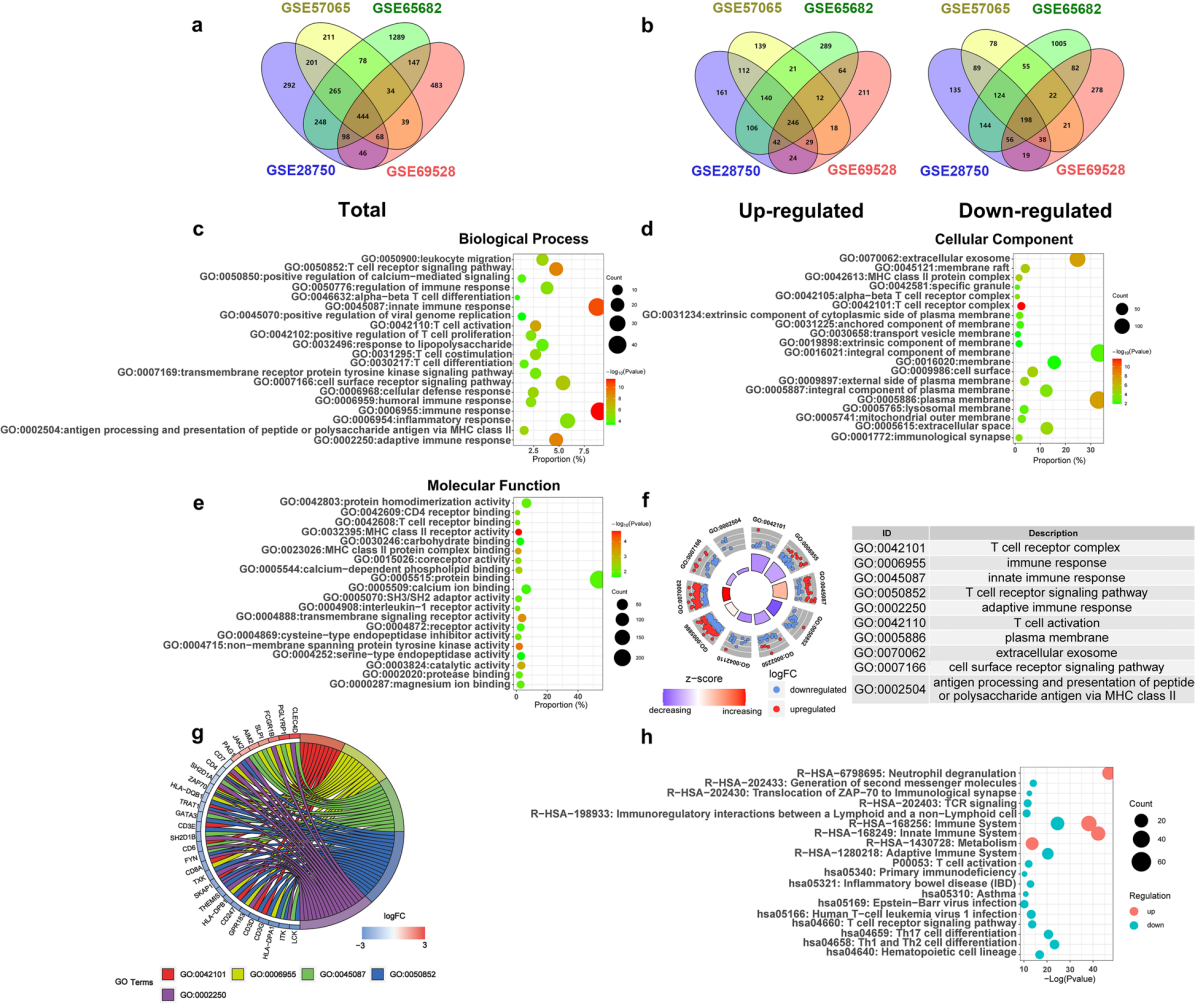
Consistent DEGs screening, GO enrichment and pathway enrichment analysis. (**A** and **B**) Identification of consistently changed DEGs from the four datasets (GSE28750, GSE57065, GSE65682 and GSE69528). The 444 common DEGs can be classified into 246 up-regulated and 198 down-regulated genes . Each color area repersented the corresponding dataset. (**C**, **D** and **E**) The results of GO analysis for the common DEGs were shown in three groups: cellular component (**C**), molecular function (**D**), and biological process (**E**). **F** The top 10 significant GO terms were shown in a GOCircle plot. The height of bars in the inner ring indicated the -log10 (*P* values) of GO terms, with higher bars representing higher significance. The colors of these bars indicated the z-score (standard score), with darker colors representing larger absolute value. The scatter plots in the out ring showed the regulation of each gene in the corresponding GO terms, with red representing up-regulated and blue representing down-regulated. The descriptions of GO categories were displayed in the table by the side. **G** The common DEGs and their linked GO terms were showed by GO chord plot. Different colors corresponding to the genes indicated different fold change levels. **H** Signaling pathway enrichment analysis for the common DEGs. DEGs, Differentially Expressed Genes. GO, Gene Ontology

The limma R package was used to screened out the DEGs. As a result, 1662, 1340, 2603 and 1359 DEGs were identified from each dataset. After integrated bioinformatical analysis, a total of 444 common DEGs were identified (Fig. 2A, Supplementary Table 2), including 246 up-regulated and 198 down-regulated genes (Fig. 2B).

To better understand biological meanings of the common DEGs, GO analysis was conducted with DAVID. The immune response, T cell receptor complex and MHC class II protein receptor activity were the most significant terms for the category of biological process, cellular component and molecular function respectively. (Fig. 2C-E). GO analysis of up-regulated and down-regulated genes was also performed separately (Supplementary Table 3). Moreover, the GOCircle plots showed the top 10 most significant GO terms (Fig. 2F), and genes involved in the the top 5 terms were exhibited using a chord plot (Fig. 2G).

Pathway enrichment analysis for the common DEGs was conducted using “KEGG PATHWAY”, “Reactome”, “Biocyc” and “Panther” databases with KOBAS 3.0 tool. Results showed that they were most significantly enriched in neutrophil deregulation and immune system (Fig. 2H). Besides, pathway analysis of up-regulated and down-regulated genes was also performed separately (Supplementary Table 4).

### Weighted Gene Co-expression Network Analysis (WGCNA) and module detection

**Fig. 3:**
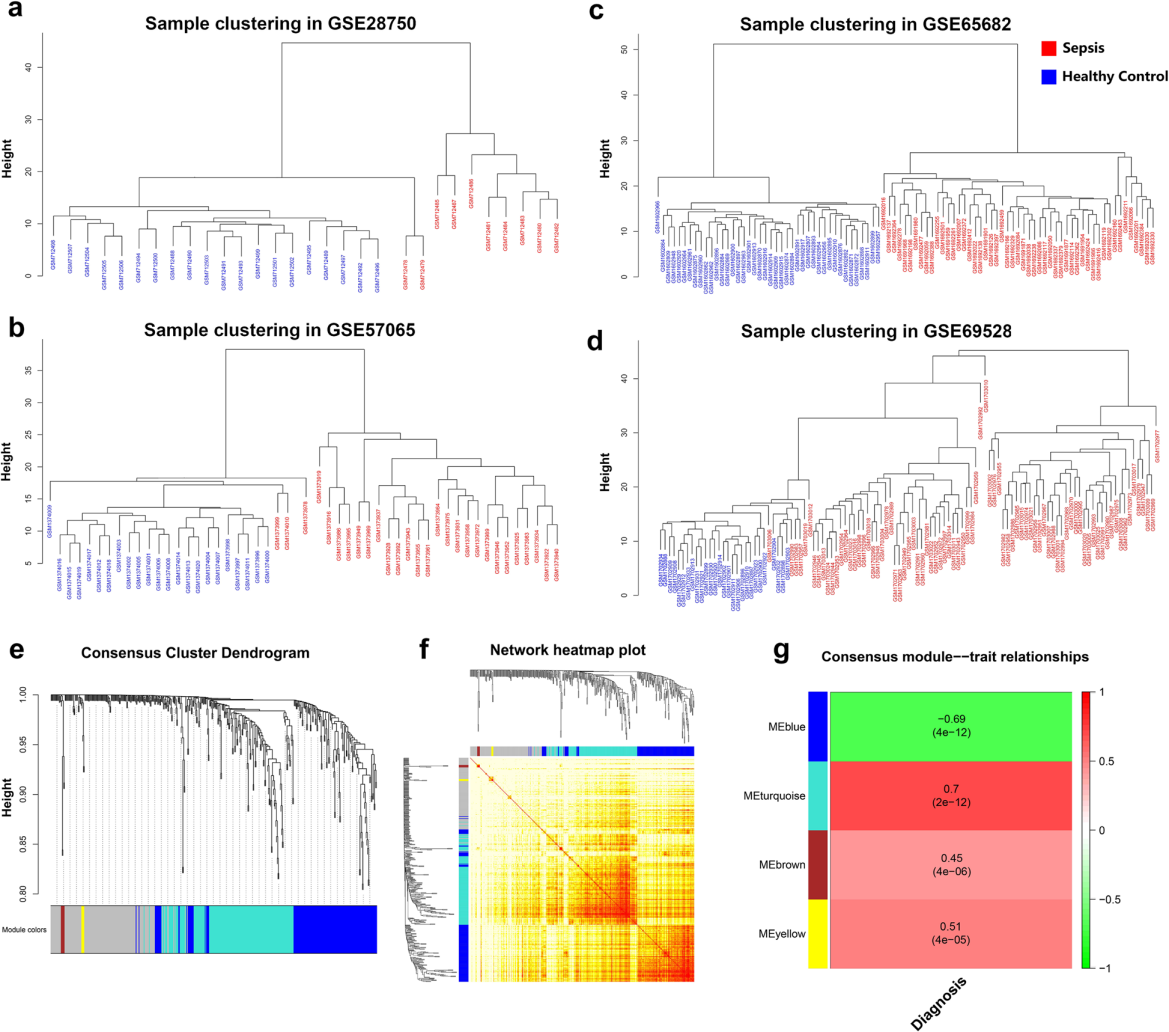
WGCNA analysis and module identification. **A-D** Sample clustering dendrogram to detect outliers in WGCNA. All samples from the four datasets (GSE28750, GSE57065, GSE65682, and GSE69528) had passed the cuts and most of the samples with the same disease were clustered together. **E** Clustering dendrograms of genes, based on topological overlap, together with assigned module colors. As a result, 4 co-expression modules were constructed and were shown in different colors. **F** The gene co-expression network was visualized in the form of heatmap. Light color represented low co-expression and progressively darker red color represented higher co-expression. The darker colors along the diagonal were the modules. **G** Module-trait associations. Each row corresponded to a module eigengene, and the column to the traits (diagnosed sepsis or healthy). Each cell contained the corresponding correlation and *p*-value. The table was color-coded by correlation according to the color legend

The gene expression matrixes of GSE28750, GSE57065, GSE65682, and GSE69528 were respectively clustered using Pearson’s correlation coefficient according to the expression profiles of the 444 common DEGs in these datasets. Clustering trees for each dataset were established and no outliers were found (Fig. 3A-D). Next, the gene modules, which represented groups of genes with similar patterns of expression, were calculated. Four gene modules were finally identified by the hierarchical clustering dendrogram. And the gray module represented genes that cannot be clustered into any other modules (Fig. 3E). Among the modules, the turquoise one was the largest, which contained many genes related to hemopoietic stem cell differentiation, such as *CD4, ITGAM* and *IL1R1*. And the blue module contained many genes such as *TRAT1*, *ZAP70*, *CD8A*, and *CD3E*, which were related to T cell activation, differentiation, receptor binding and costimulation. Therefore, this module was likely T-cell specific. Heatmap was constructed to visualize the gene co-expression network (Fig. 3F).

### Screening for clinically related modules and genes

Module eigengene is the first principal component of a given module, which can be considered a representative of the gene expression profiles in a module. The correlation between each module eigengene and clinical phenotypes was calculated [26]. The results showed that the turquoise module had the strongest association with sepsis (Fig. 3G). So, for each gene in this module, gene significance (GS) was calculated to evaluate the correlation between gene expression level and sepsis. Fifteen genes were identified according to GS value (Supplementary Table 5). And many of these genes (such as *CD177* [27, 28], *S100A12* [29, 30], and *CLEC4D* [31]) played a critical role in sepsis pathology.

### Identification of hub genes and blocks using protein-protein interaction (PPI) network

**Fig. 4:**
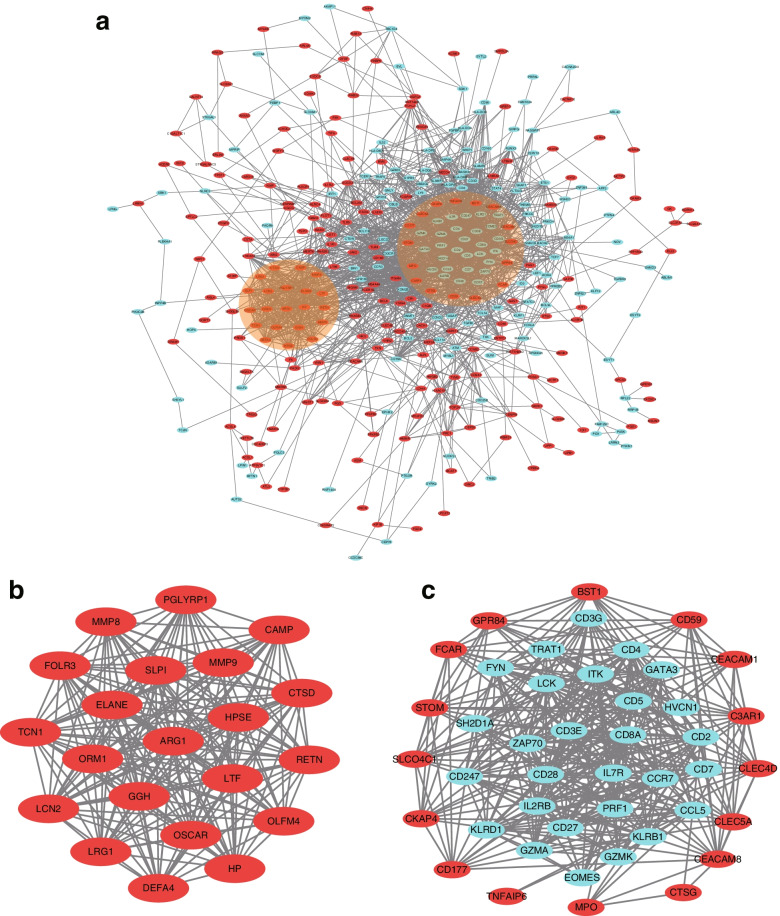
PPI network construction and significant block screening. **A** A total of 369 proteins corresponding to the common DEGs were screened out and constructed into the PPI network. The two highlighted circle areas were the most significant protein blocks. **B** and **C** were the details of the two blocks

The activity of protein-protein interactions is considered to be the prime target of cellular biology study and works as a precondition for system biology. Proteins perform their operation inside a cell with the interaction of another protein, and information that is produced from a PPI network raises perception about the function of the proteins [23]. For the reasons above, the proteins corresponding to the common DEGs were constructed into a PPI network using the STRING database (Fig. 4A). The network was composed of 369 nodes (proteins) and 2032 edges (interactions), and 75 of the 444 genes were filtered out.

Nodes that have the most interactions were considered as hub genes [23]. Among the 369 nodes, 24 were identified as the hub genes with the criteria of node degree > 35 (Supplementary Table 6), meaning that each protein expressed from these genes has more than 35 interactions. It is worth noting that many of these proteins, such as MPO [32] and CD28 [33], have been reported to play a role in sepsis. Other proteins like TLR8 could act as a potential therapeutic target [34].

Then the Molecular complex detection (MCODE) plug-in was subsequently applied to select the significant blocks in the PPI network. Two significant blocks with the highest scores were screened out. Block 1 consisted of 21 nodes and 208 edges, while block 2 was composed of 44 nodes and 371 edges (Fig. 4B-C). Notably, *ARG1* was located in the central position of block 1 (Fig. 4B).

### Identification of ARG1 as a key gene in sepsis

**Fig. 5:**
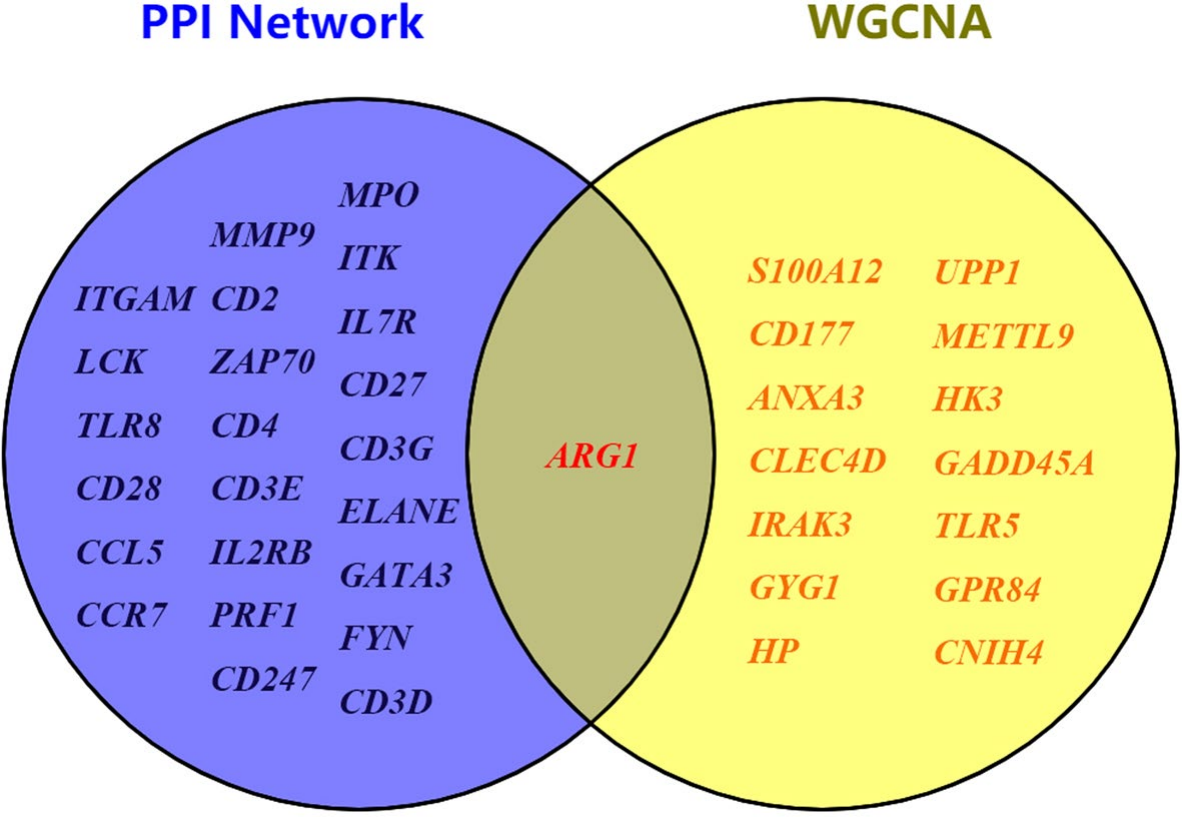
Identification of ARG1 as the key gene. The genes screened by WGCNA with the greatest GS values were compared with hub genes with more than 35 interactions identified through the PPI network. ARG1 was the only one that existed in both groups

Then genes most relevant to sepsis screened by WGCNA (Supplementary Table 5) was compared with hub genes with more than 35 interactions identified by the PPI network (Supplementary Table 6). *ARG1* was found to be the only one overlapped gene in both results (Fig. 5), indicating that this gene was not only highly correlated with the clinical phenotype of sepsis, but also played a hub role in protein-protein interactions. At the same time, *ARG1* was also located in the central position in block 1 of the PPI network (Fig. 4B). These results showed that *ARG1* was a key gene in sepsis.

### ARG1 is sharply up-regulated in the whole blood cells of septic patients

**Fig. 6:**
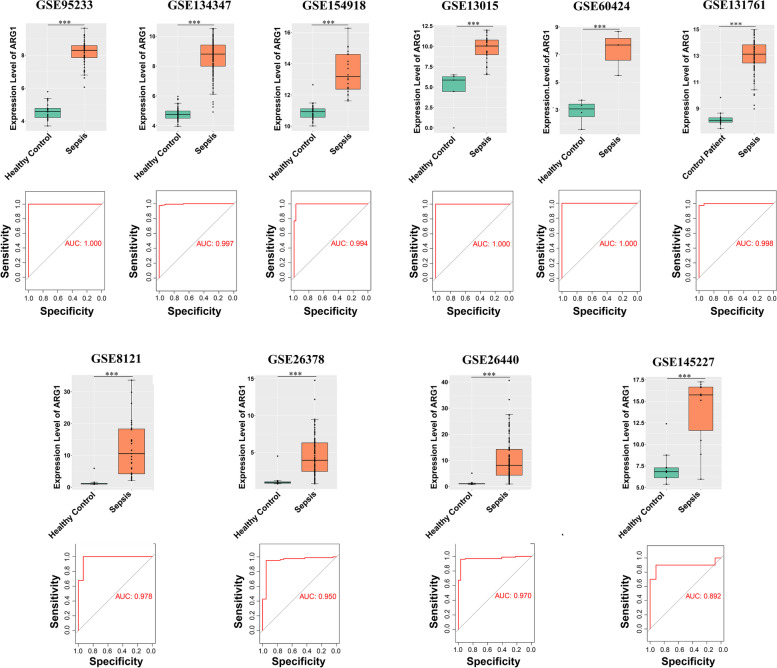
The upregulation of ARG1 in septic individuals compared to controls. The plots represented transcript abundance of ARG1 in peripheral blood, as measured by microarray or RNA-seq. The first six were conducted in adults and the following in pediatric subjects. The ROC curve for each dataset was located below the corresponding dot plot. The area under the curves (AUC) for all ROC curves were used to predict diagnostic value

In order to verify the role of *ARG1* in sepsis, more GSE datasets were brought into our analysis and validation system. The number of sepsis samples in GSE95233, GSE134347, GSE154918, GSE13015, GSE60424, GSE131761, GSE8121, GSE26378, GSE26440 and GSE145227 was 51, 156, 39, 29, 3, 81, 60, 82, 98 and 10 respectively, and the number of control samples was 22, 83, 40, 5, 4, 15, 15, 21, 32 and 12 respectively. Of these datasets: (i) Six were from studies conducted in adults and four in pediatric subjects; (ii) Five were from studies that took place in North America, four in Europe and one in Asia; (iii) Eight were performed using microarray and 2 using RNA-seq. Across 10 datasets, a significant increase in transcript abundance of *ARG1* was observed in the peripheral blood of septic patients compared with that in the control groups (Fig. 6), regardless of ethnicity, age, or experimental settings. Besides, ROC curves generated from these datasets further confirmed the role of *ARG1* in sepsis (Fig. 6). A good biomarker should exhibit high sensitivity (the fraction of correctly identified true positives) and specificity (the fraction of correctly identified true negatives), while the sensitivity and specificity are reflected by the area under the curves (AUC) value in the ROC curve. The AUC value for *ARG1* in all plots were equal or close to 1, indicating the diagnostic character of this gene (Fig. 6).

### ARG1 helps to make an accurate diagnosis, discriminate the severity and predict the treatment response of sepsis

**Fig. 7:**
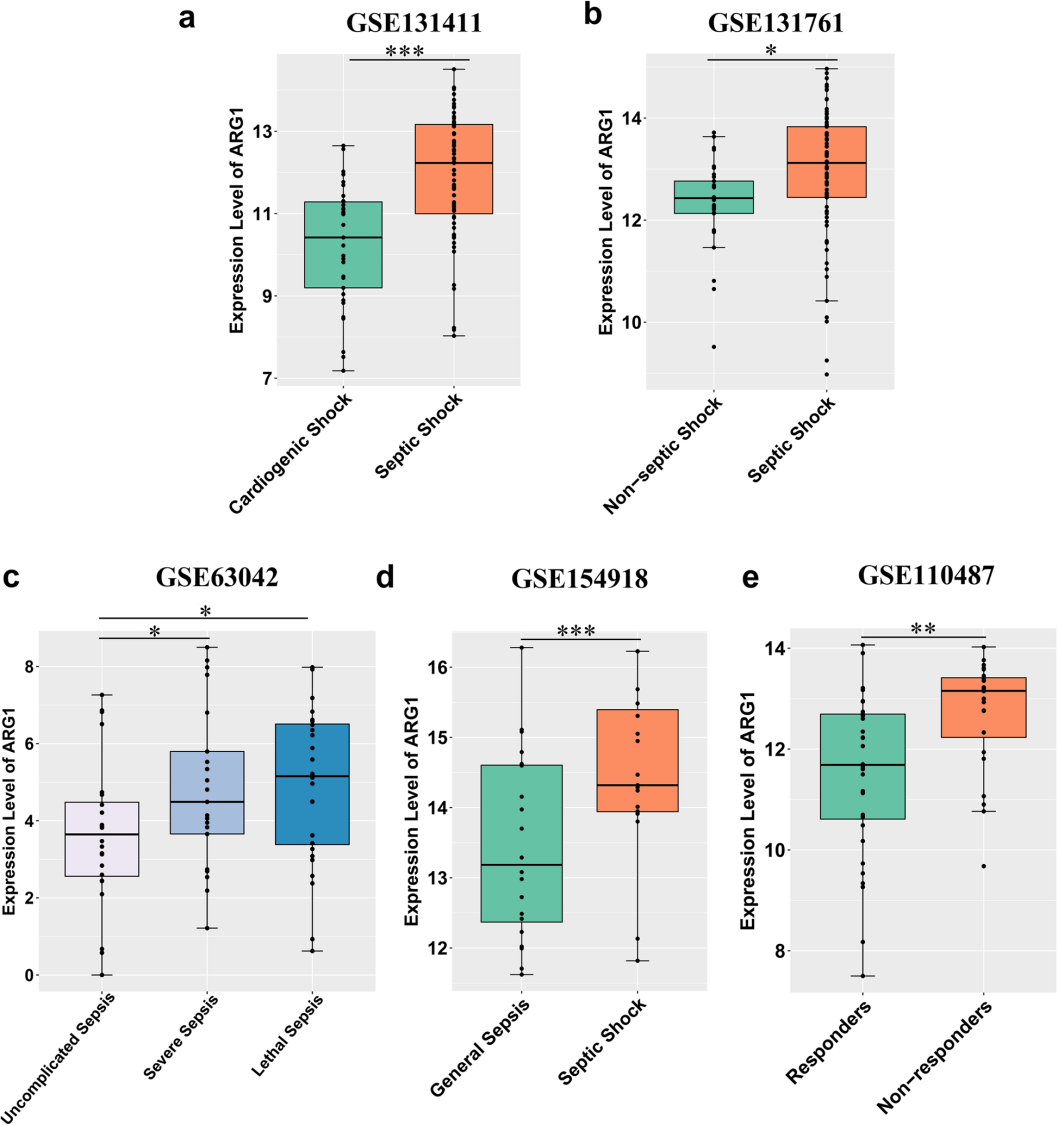
ARG1 could play a role in distinguishing sepsis from other similar diseases, predicting the response of treatment, and reflecting the severity of sepsis. **A-B** ARG1 was upregulated in peripheral blood of patients from Spain (**A**) and Italy (**B**) with septic shock compared with non-septic shock. The plots represented the transcript abundance of ARG1, as measured by microarray or RNA-seq. **C** Dataset from the USA showed the expression level of ARG1 gene in severe sepsis and lethal sepsis was significantly higher than that in uncomplicated sepsis. **D** Dataset from Germany showed ARG1 was significantly up-regulated in patients with septic shock compared with general sepsis. **E** Dataset from Italy showed a significant increase of ARG1 expression in non-responders to the early stage of treatment compared with responders

Considering the high expression of *ARG1* in sepsis, we next investigated whether *ARG1* played a role in distinguishing sepsis from diseases with similar symptoms. We only found two datasets (GSE131411 from Spain and GSE131761 from Italy) that contained peripherial blood samples from both septic and non-septic shock cases. The number of septic shock cases in GSE131411 and GSE131761 was 63 and 81 respectively, and the number of non-septic shock cases was 33 and 30 respectively. We found that the expression levels of *ARG1* were significantly higher in septic shock compared with non-septic shock (Fig. 7A-B). Since septic shock is a severe form of sepsis, and shares similar signs and symptoms with non-septic shock, it is of great value to utilize *ARG1* as a potential biomarker to distinguish the two conditions in clinical practice.

Furthermore, since the GSE63042 dataset contained 28 lethal sepsis cases, 21 severe sepsis cases, and 24 uncomplicated sepsis cases, we further revealed the role of *ARG1* in discriminating the severity of this disease. In this set of data, the expression level of *ARG1* in severe sepsis and lethal sepsis was significantly higher than that in uncomplicated sepsis (Fig. 7C). Moreover, *ARG1* expression was also found up-regulated in patients with septic shock (20 cases) compared with patients with general sepsis (19 cases) based on the dataset from Germany (Fig. 7D). These findings indicated that quantification of the expression level of *ARG1* may help to identify those at the greatest risk of progression and mortality.

Besides, our following investigations found that *ARG1* could also act as an indicator for judging whether it is responsive to early supportive therapy. In the dataset from Italy, patients received a blood check at Intensive Care Unit (ICU) admission at first, and then their responses to the early symptomatic treatment were recorded in the next few days. No significant difference was found between 32 responders and 24 non-responders regarding the source of infection, circulating markers of inflammation, or leukocyte and lymphocyte counts [35]. Interestingly, *ARG1* was high expressed in non-responders compared with responders of septic patients (*P* = 0.0017) (Fig. 7E). This finding indicated that *ARG1* may play a role in establishing the treatment response, and be helpful to predict whether early treatment for sepsis is effective.

### Validation of ARG1 as a key biomarker using quantitative real-time PCR

**Fig. 8:**
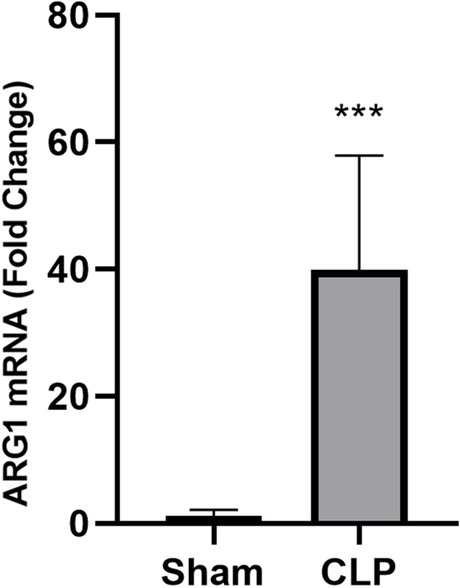
Validation of ARG1 as a key biomarker of sepsis. Real-time PCR showed that ARG1 was sharply up-regulated in CLP-induced septic mice. *N* = 7 for each group. *** *P* < 0.001

To verify the high expression of *ARG1* in sepsis, cecal ligation and puncture (CLP) was performed on mice to induce experimental sepsis. The quantitative real-time PCR showed that the transcription abundance of *AGR1* increased dramatically in the peripheral blood of septic mice (Fig. 8), demonstrating that *ARG1* is highly correlated with sepsis and have potential to act as a key biomarker.

## Discussion

In clinic, Sequential Organ Failure Assessment (SOFA) Score is now recognized as the gold standard for identifying organ dysfunction in septic patients [1]. Meanwhile, biomarkers could provide better and more rapid stratification of patients, thus help treatment. Partly due to the complexity of sepsis, it relies on a combination of biomarkers in clinical practice [6]. For example, lactate indicates tissue hypoxia, PCT indicates infection, CRP and cytokines indicate inflammation, thrombomodulin, thrombin-antithrombin complex and D-dimers indicate coagulation, angiopoietin-2 and von Willebrand Factor indicate endothelial injury, and HLA-DR on monocytes indicate immunosuppression [8, 36, 37]. Nevertheless, the mechanism of sepsis is too complex and the combination of current biomarkers is still insufficient to predict outcomes. Attributed to both the lack of specific drugs and the unsatified diagnosis, the 90-day mortality of sepsis is still as high as 32.24%.

Fortunately, in recent decades, genome-wide analysis and novel bioinformatic algorithms have been widely used to predict more precise and effective biomarkers [38]. In this study, we used PPI network and WGCNA to reveal promising biomarker genes, which may provide a supplement to classical biomarkers for septic diagnosis.

To carry out bioinformatic analysis, datasets are needed. One dataset have been used to identify core genes and pathways in diabetes mellitus [39], two datasets in cardiac-cerebral vascular disease [40], and three datasets in pancreatic cancer [41]. In this study, four cohort datasets submitted in the last 10 years were firstly used to screen the common DEGs. We chose the datasets from different countries representing different ethnicities. Later, the hub genes were identified from the DEGs by WGCNA and PPI network parallelly. Among the genes identified through the PPI network, many genes such as *MPO* [32], *CD28* [33], and *TLR8* [34] have been reported to play a vital role in sepsis. Similarly, the genes screened by WGCNA such as *CD177* [27, 28], *S100A12* [29, 30], and *CLEC4D* [31] have also been described to be important in the pathogenesis of sepsis. Notably, *ARG1* was the only overlapped gene in both results, suggesting that it may be more closely associated with sepsis.

*ARG1* encodes an arginase catalyzing the hydrolysis of arginine [42]. Through the hydrolysis of arginine by arginases, local L-arginine starvation occurs in higher vertebrates. ARG1 protein can be released from human granulocytes and maintain a very high activity in extracellular space during the inflammatory process, exerting a strong suppressible effect on immunity [43]. On the one hand, ARG1 leads to the suppression of T lymphocytes, contributing to the poor prognosis and death of septic patients [44–46]. Arginases have been shown to impaire T-cell functions by downregulating expression of T-cell receptor (TCR)-associated CD3ζ and ε chains, the critical components of the TCR-signaling complex, thereby leading to an immunosuppressive state [45]. T cell proliferation can be restored by adding arginine or arginase 1 inhibitor (such as CB-1158) to culture medium, indicating the role of ARG1 in immunosuppression [47]. On the other hand, high arginase activity leads to the down-modulation of MHC class II molecules which are necessary for antigen presentation in dendritic cells [48].

Moreover, *ARG1* is closely related to vascular dysfunction. High expression of *ARG1* may lead to local L-arginine starvation, while L-arginine is a necessary substrate of endothelial nitric oxide synthase (eNOS) in endothelial cells [49]. Through hydrolyzing arginine and disturbing eNOS activity, up-regulation of *ARG1* contributes to vasodilation dysfunction in different stages of sepsis [50]. Collectively, *ARG1* could worsen immunosuppression and vascular dysfunction during sepsis, leading to the poor prognosis, which is in accordance with our findings.

To further verify the role of *ARG1* as a key biomarker, we analyzed more datasets representing more populations. Interestingly, the transcript abundance of *ARG1* was not only higher in sepsis than that in healthy controls, but also higher in septic shock than that in non-septic shock, higher in severe or lethal sepsis than that in uncomplicated sepsis, and higher in non-responders than that in responders upon early treatment. Consistently, a meta-anslysis also reported the upregulation of *ARG1* during sepsis [51]. Our experimental results using septic mice further verified the upregulation of *ARG1* in the peripheral blood cells of septic animals. All these informations indicated the potential of *ARG1* as a biomarker in accurate diagnosis, prediction and treatment of sepsis. Since the transcription level of *ARG1* is significantly high in sepsis, Q-PCR could be a promising method for rapid test of ARG1 as a biomarker.

In conclusion, our study showed *ARG1* could act as a potential “multifunctional” biomarker to provide more information for the diagnosis of sepsis, prediction of severity, and judgement of the responsiveness to supportive therapy. Besides, this study provided a novel strategy to identify biomarkers by looking for the common genes screened by PPI network and WGCNA.

## Supplementary Information

**Additional file 1: Supplementary Table 1.** Details of the datasets used in this study. **Supplementary Table 2.** Shared differentially expressed genes (DEGs) from the datasets GSE28750, GSE57065, GSE65682 and GSE69528. **Supplementary Table 3.** Results of GO analysis for the up-regulated and down-regulated shared DEGs. **Supplementary Table 4.** Results of pathway analysis for the up-regulated and down-regulated shared DEGs. **Supplementary Table 5.** Genes with greatest GS value in the turquoise module. **Supplementary Table 6.** Key candidate genes with node degree >35 identified by PPI network.

## Data Availability

Data used to perform bioinformatic analysis are available from the public databases. And the experimental data in the current study are available from the corresponding author on reasonable request.

## Abbreviations

ARG1: Arginase 1
AUC: Area Under the Curves
CLP: Cecum Ligation and Puncture
CRP: C-reactive Protein
DAVID: Database for Annotation, Visualization and Integrated Discovery
DEGs: Differentially Expressed Genes
FC: Fold Change
GAPDH: Glyceraldehyde-3-Phosphate Dehydrogenase
GEO: Gene Expression Omnibus
GO: Gene Oncology
GS: Gene Significance Value
HLA-DR: Human Leukocyte Antigen DR
KOBAS: KEGG Orthology Based Annotation System
MCODE: Molecular Complex Detection
ME: Module Eigengene
PCT: Proclcitonin
PPI Network: Protein-Protein Interaction Network
ROC: Receiver Operating Characteristic
STRING: Search Tool for the Retrieval of Interacting Genes/Proteins
WGCNA: Weighted Gene Correlation Network Analysis
