# Supplementary material for: ARG1 as a promising biomarker for sepsis diagnosis and prognosis: evidence from WGCNA and PPI network
Source: Hereditas. 2022 Jun 23;159:27. doi: 10.1186/s41065-022-00240-1 (PMC9219214; doi:10.1186/s41065-022-00240-1)
Supplement: Supplementary file 1 — Additional file 1: Supplementary Table 1. Details of the datasets used in this study. Supplementary Table 2. Shared differentially expressed genes (DEGs) from the datasets GSE28750, GSE57065, GSE65682 and GSE69528. Supplementary Table 3. Results of GO analysis for the up-regulated and down-regulated shared DEGs. Supplementary Table 4. Results of pathway analysis for the up-regulated and down-regulated shared DEGs. Supplementary Table 5. Genes with greatest GS value in the turquoise module. Supplementary Table 6. Key candidate genes with node degree >35 identified by PPI network. [file 41065_2022_240_MOESM1_ESM.docx]

Supplementary Table 1. Details of the datasets used in this study

| **Peripheral Blood from Sepsis Patients v.s. Healthy Controls** | | | | | | | |
| --- | --- | --- | --- | --- | --- | --- | --- |
| Dataset ID | Technology | Platform | Number of sepsis samples | Number of healthy control samples | Country | Citation Pubmed ID (PMID) | Remarks |
| GSE8121 | Microarray | GPL570 | 60 | 15 | USA | 17932561 | Pediatric Sepsis |
| GSE13015 | Microarray | GPL6106 | 29 | 5 | USA | 19903332 |  |
| GSE26378 | Microarray | GPL570 | 82 | 21 | USA | 21738952 | Pediatric Sepsis |
| GSE26440 | Microarray | GPL570 | 98 | 32 | USA | 19624809 | Pediatric Sepsis |
| GSE28750 | Microarray | GPL570 | 10 | 20 | Australia | 21682927 |  |
| GSE57065 | Microarray | GPL570 | 26 | 25 | France | 26215705 |  |
| GSE60424 | RNA Sequencing | GPL15456 | 3 | 4 | USA | 25314013 |  |
| GSE65682 | Microarray | GPL13667 | 51 | 42 | Malta | 26121490 |  |
| GSE69528 | Microarray | GPL10558 | 83 | 28 | USA | 19903332 |  |
| GSE95233 | Microarray | GPL570 | 51 | 22 | France | 28341250 |  |
| GSE134347 | Microarray | GPL17586 | 156 | 83 | Malta | 33305733 |  |
| GSE145227 | Microarray | GPL23178 | 10 | 12 | China | 32151258 | Pediatric Sepsis |
| GSE154918 | RNA Sequencing | GPL20301 | 39 | 40 | Germany | 33640018 |  |
| **Peripheral Blood from Sepsis Patients v.s. Control Patients** | | | | | | | |
| Dataset ID | Technology | Platform | Number of sepsis samples | Number of control samples | Country | Citation Pubmed ID (PMID) | Remarks |
| GSE131761 | Microarray | GPL13497 | 81 | 15 | Spain | 34144116 |  |
| **Peripheral Blood from Septic Shock Patients v.s. Non-septic Shock Patients** | | | | | | | |
| Dataset ID | Technology | Platform | Number of septic shock samples | Number of non-septic shock samples | Country | Citation Pubmed ID (PMID) | Remarks |
| GSE131411 | RNA Sequencing | GPL10999  GPL16791 | 63 | 33 | Italy | 31856860 |  |
| GSE131761 | Microarray | GPL13497 | 81 | 33 | Spain | 34144116 |  |
| **Responders to Supportive Therapy v.s. Non-responders to Supportive Therapy** | | | | | | | |
| Dataset ID | Technology | Platform | Number of samples from responders | Number of samples from non-responders | Country | Citation Pubmed ID (PMID) | Remarks |
| GSE110487 | RNA Sequencing | GPL10999  GPL16791 | 32 | 24 | Italy | 30463588 |  |
| **General Sepsis v.s. Severe or Lethal Sepsis** | | | | | | | |
| Dataset ID | Technology | Platform | Number of samples from general sepsis | Number of samples from severe or lethal sepsis | Country | Citation Pubmed ID (PMID) | Remarks |
| GSE63042 | RNA Sequencing | GPL9115 | 24 | 49 | USA | 25538794 |  |
| GSE154918 | RNA Sequencing | GPL20301 | 20 | 19 | Germany | 33640018 |  |

Supplementary Table 2. Shared differentially expressed genes (DEGs) from the datasets GSE28750, GSE57065, GSE65682 and GSE69528

| **DEGs** | **Gene Name (Official Gene Symbol)** |
| --- | --- |
| Up-regulated | *MMP8, CD177, RETN, MS4A4A, ARG1, HP, OLFM4, VNN1, LCN2, CEACAM8, TDRD9, OLAH, CLEC4D, ANXA3, GPR84, LTF, ANKRD22, IL1R2, CYP1B1, SMPDL3A, METTL7B, S100A12, CLEC5A, IRAK3, GRB10, PFKFB2, ZDHHC19, TCN1, DACH1, FAM20A, BCL2A1, PFKFB3, ADAM9, SAMSN1, GADD45A, HPGD, GYG1, DEFA4, IL18R1, UGCG, RNASE2, BMX, MMP9, STOM, PCOLCE2, CA4, CNIH4, NAIP, PDGFC, RGL4, ACER3, KCNE1, NLRC4, CEACAM1, HK3, TLR5, PLSCR1, FCGR1B, SORT1, CR1, ELANE, CKAP4, LY96, FAR2, ATP6V1C1, HMGB2, FCAR, SERPINB1, ACSL4, NSUN7, FOLR3, KLHL2, CD163, ATP9A, CST7, KIAA0101, SOCS3, CEACAM6, ST6GALNAC3, RNASE3, FGD4, SULT1B1, ELL2, MCTP1, AGFG1, CDK5RAP2, TNFAIP6, KIF1B, F5, TMTC1, IDI1, LILRA5, BCAT1, LIN7A, UPP1, HMMR, OPLAH, FKBP5, MEF2A, RAB13, IL1R1, PGLYRP1, METTL9, PLBD1, CCPG1, TMEM165, IL18RAP, GPR160, SLC25A40, CD59, CPEB4, PSTPIP2, LRRN1, AIM2, SIPA1L2, CCNA1, BST1, SLC26A8, PGS1, SLCO4C1, SLC39A8, TXN, MCTP2, DDAH2, LDHA, TP53I3, TOP2A, CD55, MAPK14, ENTPD7, C3AR1, C1QB, MGST1, EXOSC4, WDFY3, MMRN1, CARD6, ALPL, TSHZ3, GALNT14, WIPI1, MTHFD2, LPCAT2, GGH, FBN2, ORM1, MPO, TSPO, CLEC1B, PROS1, SPCS3, ITGAM, GAS7, PADI4, EMILIN2, C5orf30, B4GALT5, EXOC6, DAAM2, SLC37A3, FCER1G, MGAM, PGD, LRG1, TMCO3, TMEM167A, CMTM4, SAP30, SRPK1, FBXO30, HIST1H2BD, BCL6, DRAM1, ACSL1, NEDD4, C1GALT1C1, SLC22A4, PYGL, PRC1, OSCAR, JAK2, MERTK, LTB4R, KREMEN1, PHTF1, DYSF, S100P, ERLIN1, PAG1, CLEC4E, HPSE, PDSS1, CEP55, ADM, ETS2, DSC2, PPP1R3D, CACNA1E, UPB1, CCNB2, MKNK1, BLOC1S1, ZNF438, SH3GLB1, RBMS1, RNF144B, DIRC2, SLPI, PECR, SPTLC2, RAB32, MTF1, CARD16, CENPW, GM2A, RIT1, SLC22A15, GK, TIFA, TYMS, VSIG4, ECHDC3, CDC42EP3, VNN2, PXK, RRAGD, FLOT1, TLR8, ROPN1L, GPR141, ARL4A, ACVR1B, CSTA, CTSG, ENTPD1, MANSC1, CAMP, BEX1, SLC36A1, NQO2, CTSD, ATL3, TPST1, MYL6B, SIGLEC5, PADI2* |
| Down-regulated | *SBK1, PRSS33, TC2N, ZNF275, EPHX2, ZNF827, FCRLA, KIAA1147, MYOM2, PIK3C2B, FAM43A, DHRS3, SLAMF6, AKAP11, FLT3LG, MTX3, PQLC3, LPIN1, RNF144A, KLF12, ATM, BACH2, BTN3A2, CBLB, NUCKS1, BCL11A, CEP78, KIAA0355, MAP4K1, MPRIP, LFNG, SMAD3, RPL22, ADARB1, KLRD1, THEMIS, NFATC3, RSAD1, UBE2Q2, CXXC5, PPP1R16B, BCL9L, ARL4C, CD5, ESYT2, SULF2, CD6, TAGAP, DCAF16, PEBP1, CX3CR1, SLC38A1, WWP1, SUN1, RRP1B, CLEC2D, MAF, HOPX, PTPN4, BIN1, ST6GAL1, SYTL2, FAM129C, TGFBR3, HVCN1, INPP4B, DYRK2, HLA-DQB1, ZNF266, NMT2, SLC7A6, PRR5L, KDM2B, CACNA2D3, ZNF573, CCL5, PRKCQ, SLAMF1, HSH2D, FYN, HLA-DOB, RNF125, ZNF529, PID1, FAM117B, CCDC88C, ETS1, MS4A1, PRF1, ZNF83, HLA-DPA1, LY9, DNMT1, PIK3IP1, GOLGA8A, RPS6KA5, TCF7, BCL2, HLA-DOA, ZBTB4, GATA3, ZNF91, ESYT1, CCR6, ID3, SKAP1, CCND2, ZSCAN18, PLEKHA1, AUTS2, PAQR8, VPREB3, CD4, ZNF395, S1PR5, CDC25B, CTSW, STAT4, RFTN1, P2RY10, DOCK10, TGFBI, TBC1D4, TCL1A, RORA, ZNHIT6, SIRPG, ZAP70, MAN1C1, CD79A, SGK1, HLA-DMA, GZMH, CD96, GZMA, CD160, SIDT1, EVL, AFF3, HLA-DMB, TRAT1, SH2D1A, PYHIN1, SPOCK2, MAL, CD27, PTGDR, MARCKSL1, CD7, LEF1, GPR183, TRIB2, CD3D, RUNX3, CD28, CD8A, EOMES, GZMK, PASK, KLRB1, FAM102A, PRKCH, LCK, HLA-DPB1, SAMD3, RASGRP1, NOV, LBH, MYBL1, NLRC3, KLRF1, SH2D1B, IL7R, UBASH3A, TXK, SGK223, ATP8B2, FGFBP2, IL32, IFIT1, CD3G, TRAF5, ABLIM1, IL2RB, ITK, CLIC3, LRRN3, CD2, NELL2, CCR7, BCL11B, GPR18, SH3YL1, CD3E, CD247, CCR3, GNLY, FCER1A* |

Supplementary Table 3. Results of GO analysis for the up-regulated and down-regulated shared DEGs

| **Regulation** | **Term** | **Description** | **Category** | **Count** | **P-Value** |
| --- | --- | --- | --- | --- | --- |
| Up | GO:0070062 | extracellular exosome | CC | 84 | 6.05×10^-14^ |
|  | GO:0045087 | innate immune response | BP | 26 | 2.66×10^-09^ |
|  | GO:0005615 | extracellular space | CC | 44 | 3.36×10^-08^ |
|  | GO:0019731 | antibacterial humoral response | BP | 7 | 3.50×10^-05^ |
|  | GO:0042742 | defense response to bacterium | BP | 11 | 4.35×10^-05^ |
|  | GO:0003824 | catalytic activity | MF | 12 | 5.74×10^-05^ |
|  | GO:0050900 | leukocyte migration | BP | 10 | 6.23×10^-05^ |
|  | GO:0031225 | anchored component of membrane | CC | 9 | 1.15×10^-04^ |
|  | GO:0032496 | response to lipopolysaccharide | BP | 11 | 1.23×10^-04^ |
|  | GO:0006954 | inflammatory response | BP | 16 | 3.59×10^-04^ |
| Down | GO:0050852 | T cell receptor signaling pathway | BP | 20 | 4.57×10^-15^ |
|  | GO:0042101 | T cell receptor complex | CC | 10 | 5.66×10^-14^ |
|  | GO:0006955 | immune response | BP | 27 | 1.64×10^-12^ |
|  | GO:0042110 | T cell activation | BP | 11 | 9.27×10^-11^ |
|  | GO:0002250 | adaptive immune response | BP | 16 | 1.20×10^-10^ |
|  | GO:0031295 | T cell costimulation | BP | 12 | 1.11×10^-09^ |
|  | GO:0009897 | external side of plasma membrane | CC | 16 | 7.40×10^-09^ |
|  | GO:0050776 | regulation of immune response | BP | 15 | 1.40×10^-08^ |
|  | GO:0002504 | antigen processing and presentation of peptide or polysaccharide antigen via MHC class II | BP | 7 | 2.10×10^-08^ |
|  | GO:0042613 | MHC class II protein complex | CC | 7 | 7.95×10^-08^ |

Supplementary Table 4. Results of pathway analysis for the up-regulated and down-regulated shared DEGs

| **A. Upregulated DEGs** | | | | |
| --- | --- | --- | --- | --- |
| **Pathway** | **Name** | **Gene Count** | **P-Value** | **Genes** |
| Reactome: R-HSA-6798695 | Neutrophil degranulation | 53 | 1.32×10^-47^ | *ELANE, TNFAIP6, CD55, CD59, S100P, CD177, MAPK14, STOM, PGLYRP1, MGST1, OLFM4, S100A12, GYG1, CLEC5A, FCER1G, C3AR1, OSCAR, MGAM, LCN2, RETN, CEACAM1, SLCO4C1, DEFA4, CEACAM6, CEACAM8, GPR84, GGH, FCAR, HPSE, RNASE3, RNASE2, VNN1, CTSD, CTSG, PADI2, LTF, SIGLEC5, SERPINB1, LRG1, SLPI, CR1, ORM1, PYGL, GM2A, TCN1, CLEC4D, BST1, ITGAM, MMP8, MMP9, MPO, CKAP4, FOLR3* |
| Reactome: R-HSA-168249 | Innate Immune System | 64 | 6.03×10^-43^ | *ELANE, GM2A, CD55, CD59, PROS1, S100P, TLR5, CD177, MAPK14, LY96, PGLYRP1, MGST1, OLFM4, NLRC4, S100A12, GYG1, IRAK3, CLEC5A, FCER1G, C3AR1, OSCAR, MGAM, LCN2, RETN, CEACAM1, SLCO4C1, DEFA4, CEACAM6, CEACAM8, GPR84, GGH, FCAR, HPSE, RNASE3, RNASE2, VNN1, CTSD, AIM2, PADI2, LTF, SIGLEC5, SERPINB1, LRG1, CTSG, TLR8, SLPI, CR1, ORM1, STOM, PYGL, CLEC4E, TNFAIP6, TCN1, C1QB, CLEC4D, MEF2A, BST1, ITGAM, MMP8, MMP9, MPO, ATP6V1C1, CKAP4, FOLR3* |
| Reactome: R-HSA-168256 | Immune System | 79 | 6.75×10^-39^ | *BST1, ELANE, GM2A, CD55, PAG1, CD59, PROS1, S100P, TLR5, CD177, MAPK14, LY96, PGLYRP1, MGST1, FCGR1B, OLFM4, NLRC4, S100A12, GYG1, IL1R2, IL1R1, IRAK3, CLEC5A, FCER1G, C3AR1, IL18RAP, OSCAR, MGAM, LCN2, NEDD4, RETN, CEACAM1, SLCO4C1, DEFA4, JAK2, CEACAM6, CEACAM8, GPR84, GGH, FCAR, HPSE, RNASE3, RNASE2, VNN1, CTSD, IL18R1, TIFA, AIM2, CR1, KLHL2, LTF, SIGLEC5, SERPINB1, RNF144B, LRG1, CTSG, TLR8, SLPI, LILRA5, ORM1, SOCS3, STOM, PYGL, CLEC4E, TNFAIP6, TCN1, C1QB, CLEC4D, MEF2A, PADI2, FBXO30, ITGAM, MMP8, MMP9, MPO, ATP6V1C1, BCL6, CKAP4, FOLR3* |
| Reactome: R-HSA-1430728 | Metabolism | 47 | 2.89×10^-14^ | *ENTPD7, UGCG, ACSL1, SULT1B1, IDI1, NQO2, PECR, MGST1, UPB1, GYG1, OPLAH, TXN, PGD, CYP1B1, ENTPD1, GK, OLAH, CACNA1E, HPGD, ACER3, LPCAT2, SPTLC2, BMX, B4GALT5, LDHA, HMMR, PLBD1, TPST1, VNN2, VNN1, BCAT1, PDSS1, TSPO, HPSE, UPP1, MTHFD2, PFKFB2, PYGL, PFKFB3, GM2A, TCN1, ACSL4, CA4, TYMS, BST1, FAR2, DDAH2* |
| KEGG PATHWAY: hsa01100 | Metabolic pathways | 32 | 8.40×10^-10^ | *UGCG, PFKFB2, IDI1, MGST1, UPB1, GYG1, OPLAH, PGD, GK, OLAH, LPCAT2, SPTLC2, B4GALT5, LDHA, ENTPD1, UPP1, BCAT1, ST6GALNAC3, HPSE, GALNT14, MTHFD2, PYGL, PFKFB3, ACSL1, C1GALT1C1, ACSL4, CA4, TYMS, BST1, ATP6V1C1, MGAM, ALPL* |
| KEGG PATHWAY: hsa04610 | Complement and coagulation cascades | 9 | 4.54×10^-09^ | *CR1, F5, CD55, C3AR1, VSIG4, CD59, PROS1, C1QB, ITGAM* |
| Reactome: R-HSA-977606 | Regulation of Complement cascade | 7 | 4.61×10^-08^ | *ELANE, CR1, CD55, C3AR1, CD59, PROS1, C1QB* |
| Reactome: R-HSA-1474244 | Extracellular matrix organization | 13 | 9.53×10^-08^ | *ELANE, PCOLCE2, EMILIN2, CTSD, CEACAM6, FBN2, ADAM9, CEACAM1, ITGAM, MMP8, MMP9, CEACAM8, CTSG* |
| Reactome: R-HSA-166658 | Complement cascade | 7 | 1.53×10^-07^ | *ELANE, CR1, CD55, C3AR1, CD59, PROS1, C1QB* |
| KEGG PATHWAY: hsa05202 | Transcriptional misregulation in cancer | 10 | 4.86×10^-07^ | *ELANE, BCL2A1, MPO, CCNA1, HPGD, ITGAM, MMP9, GADD45A, BCL6, IL1R2* |
| **B. Downregulated DEGs** | | | | |
| **Pathway** | **Name** | **Gene Count** | **P-Value** | **Genes** |
| Reactome: R-HSA-168256 | Immune System | 56 | 2.68×10^-25^ | *IL7R, RASGRP1, BTN3A2, KLRD1, WWP1, SH2D1B, SH2D1A, SMAD3, CD4, CD8A, NFATC3, NLRC3, CCR6, EVL, HLA-DMB, IFIT1, CD79A, PEBP1, STAT4, FCER1A, TRAT1, FYN, RORA, CCL5, HLA-DQB1, ZAP70, MS4A1, KLRB1, HLA-DPB1, CD28, FLT3LG, GNLY, CD27, KLRF1, IL32, PRKCQ, TXK, GATA3, PTPN4, UBE2Q2, IL2RB, RPS6KA5, ITK, CD96, CD160, HVCN1, CLEC2D, CD3E, HLA-DOA, HLA-DOB, HLA-DPA1, SLAMF6, RNF125, CD3D, LCK, BCL2* |
| KEGG PATHWAY: hsa04658 | Th1 and Th2 cell differentiation | 19 | 5.45×10^-24^ | *HLA-DMB, HLA-DPB1, IL2RB, NFATC3, CD3E, HLA-DPA1, CD3D, LCK, CD4, RUNX3, STAT4, HLA-DOA, HLA-DOB, MAF, PRKCQ, ZAP70, CD247, GATA3, HLA-DMA* |
| KEGG PATHWAY: hsa04659 | Th17 cell differentiation | 18 | 2.32×10^-21^ | *HLA-DPB1, IL2RB, NFATC3, LCK, CD3E, HLA-DMB, RORA, SMAD3, CD4, HLA-DPA1, CD247, HLA-DOA, HLA-DOB, PRKCQ, ZAP70, CD3D, GATA3, HLA-DMA* |
| Reactome: R-HSA-1280218 | Adaptive Immune System | 33 | 4.44×10^-21^ | *KLRB1, BTN3A2, KLRD1, WWP1, SH2D1B, SH2D1A, CD4, CD8A, RASGRP1, EVL, HLA-DMB, KLRF1, TRAT1, FYN, NFATC3, ZAP70, HLA-DQB1, CD79A, HLA-DPB1, CD28, PRKCQ, UBE2Q2, ITK, CD96, CD160, CLEC2D, LCK, HLA-DOA, HLA-DOB, HLA-DPA1, SLAMF6, CD3D, CD3E* |
| KEGG PATHWAY: hsa04640 | Hematopoietic cell lineage | 15 | 1.66×10^-17^ | *IL7R, HLA-DPB1, CD2, HLA-DMB, CD4, CD5, CD7, FLT3LG, HLA-DOA, HLA-DOB, CD8A, HLA-DPA1, CD3D, CD3E, HLA-DMA* |
| Reactome: R-HSA-202433 | Generation of second messenger molecules | 10 | 9.34×10^-15^ | *HLA-DPB1, EVL, ITK, LCK, CD4, HLA-DQB1, ZAP70, HLA-DPA1, CD3D, CD3E* |
| KEGG PATHWAY: hsa04660 | T cell receptor signaling pathway | 13 | 2.65×10^-14^ | *RASGRP1, ITK, FYN, CD3E, CD4, CD8A, CD28, CD247, NFATC3, ZAP70, PRKCQ, CD3D, LCK* |
| KEGG PATHWAY: hsa05166 | Human T-cell leukemia virus 1 infection | 16 | 7.06×10^-14^ | *HLA-DPB1, IL2RB, NFATC3, CD3E, ATM, HLA-DMB, CCND2, SMAD3, CD4, ETS1, HLA-DOA, HLA-DOB, HLA-DPA1, CD3D, LCK, HLA-DMA* |
| KEGG PATHWAY: hsa05321 | Inflammatory bowel disease (IBD) | 11 | 1.49×10^-13^ | *HLA-DPB1, STAT4, RORA, SMAD3, HLA-DOA, HLA-DOB, MAF, HLA-DPA1, HLA-DMB, GATA3, HLA-DMA* |
| Reactome: R-HSA-202430 | Translocation of ZAP-70 to Immunological synapse | 8 | 4.66×10^-13^ | *HLA-DPB1, CD3E, CD4, HLA-DQB1, ZAP70, HLA-DPA1, CD3D, LCK* |

Supplementary Table 5. Genes with greatest GS value in the turquoise module

| **Gene** | **Gene Significance Value** | **P-Value** |
| --- | --- | --- |
| *S100A12* | 23.67 | 7.37×10^-124^ |
| *CD177* | 23.53 | 2.16×10^-122^ |
| *ANXA3* | 23.33 | 2.46×10^-120^ |
| *CLEC4D* | 23.31 | 3.48×10^-120^ |
| *IRAK3* | 23.24 | 1.90×10^-119^ |
| *GYG1* | 22.85 | 1.62×10^-115^ |
| *HP* | 22.55 | 1.31×10^-112^ |
| *UPP1* | 22.38 | 5.85×10^-111^ |
| *METTL9* | 21.76 | 5.73×10^-105^ |
| *HK3* | 21.74 | 8.93×10^-105^ |
| *GADD45A* | 21.60 | 1.79×10^-103^ |
| *TLR5* | 21.59 | 2.09×10^-103^ |
| *ARG1* | 21.57 | 3.38×10^-103^ |
| *GPR84* | 21.44 | 6.20×10^-102^ |
| *CNIH4* | 21.16 | 2.27×10^-99^ |

Supplementary Table 6. Key candidate genes with node degree >35 identified by PPI network

| **Gene** | **Degree** | **Gene** | **Degree** |
| --- | --- | --- | --- |
| ITGAM | 78 | PRF1 | 42 |
| LCK | 53 | CD247 | 42 |
| TLR8 | 50 | FYN | 41 |
| CD28 | 50 | CD3D | 39 |
| CCL5 | 49 | MPO | 39 |
| CCR7 | 47 | ITK | 39 |
| MMP9 | 47 | IL7R | 38 |
| CD2 | 47 | CD27 | 38 |
| ZAP70 | 46 | CD3G | 37 |
| CD4 | 42 | ARG1 | 37 |
| CD3E | 42 | ELANE | 37 |
| IL2RB | 42 | GATA3 | 37 |
